# Treatment rationale and design of the SPIRAL study: A phase II trial of osimertinib in elderly epidermal growth factor receptor T790M-positive nonsmall-cell lung cancer patients who progressed during prior EGFR-TKI treatment

**DOI:** 10.1097/MD.0000000000011081

**Published:** 2018-06-18

**Authors:** Junji Uchino, Akira Nakao, Nobuyo Tamiya, Yoshiko Kaneko, Tadaaki Yamada, Kenichi Yoshimura, Masaki Fujita, Koichi Takayama

**Affiliations:** a Department of Pulmonary Medicine, Kyoto Prefectural University of Medicine, Kyoto; b Department of Respiratory Medicine, Faculty of Medicine, Fukuoka University, Fukuoka; c Department of Biostatistics, Innovative Clinical Research Center, Kanazawa University, Kanazawa, Ishikawa, Japan.

**Keywords:** Elderly, NSCLC, Osimertinib, T790M

## Abstract

**Background:**

Advances in epidermal growth factor receptor-tyrosine kinase inhibitor (EGFR-TKI) treatment led to research on the mechanism of the resistance have revealed that an occurrence of T790M gene mutation generated in exon 20 of the EGFR gene is associated with approximately 50% to 60% of observed resistance. Osimertinib, a 3rd-generation EGFR-TKI, has been shown to be effective against both EGFR tyrosine kinase inhibitor-sensitizing and T790M resistance mutations. In this study, we prospectively investigate the efficacy and safety of osimertinib in elderly patients aged ≥75 years, with ineffective prior EGFR-TKI treatment or with recurrence of EGFR-TKI mutation-positive or T790M mutation-positive nonsmall-cell lung cancer.

**Patients and methods:**

In total, 35 subjects of both sexes aged ≥75 years with T790M mutation will be included. Participants with pulmonary disorders such as idiopathic pulmonary fibrosis, interstitial pneumonia, pneumoconiosis, active radiation pneumonitis, drug-induced pneumonia, and symptomatic brain metastasis will be excluded. Eligible patients will be administrated osimertinib (80 mg/d) until disease progression. The primary outcome is antitumor effect (objective response rate). The secondary outcomes are progression-free survival, overall survival, disease control rate, and safety.

**Ethics and dissemination:**

The protocol was approved by the institutional review boards of Kyoto Prefectural University of Medicine and all the participating hospitals. Written informed consent was obtained from all patients before registration, in accordance with the Declaration of Helsinki. Results of the study will be disseminated via publications in peer-reviewed journals.

**Trial registration:**

Trial registration number = UMIN000022553.

## 1 Introduction

In recent years, advances in epidermal growth factor receptor-tyrosine kinase inhibitor (EGFR-TKI) treatment led to research on the mechanism of the resistance have revealed that an occurrence of T790M gene mutation generated in exon 20 of the EGFR gene is associated with approximately 50% to 60% of observed resistance.^[1]^

Afatinib, a 2nd-generation EGFR-TKI covalently linked in a tyrosine kinase domain of EGFR, is known to show a high antitumor effect by inhibiting phosphorylation irreversibly, and there was a report that showed a proliferation inhibitory effect on lung cancer cell strain that had T790M mutation^[2]^; therefore, therapeutic effects in patients with T790M mutation-positive lung cancer (T790M-positive patients) were initially expected. However, at present there has been no study in which a primary endpoint was achieved in a clinical trial for T790M-positive patients. Moreover, it is known that digestive organ toxicity and dermal toxicity of afatinib are strong, and as for the safety in the elderly, the medical evidence is not yet clear, unlike that for the 1st-generation EGFR-TKI.

Osimertinib is a 3rd-generation EGFR-TKI. Osimertinib has potential inhibitory effect on not only mutant form tyrosine kinase sensitive to 1st- and 2nd-generation EGFR-TKI, but also T790M mutant form EGFR tyrosine kinase, and has high selectivity for low inhibitory activity on wild-type EGFR tyrosine kinase.^[3]^ When the inhibitory activity on wild-type EGFR-TKI is high, toxicity such as rash and diarrhea is observed. Thus, it is thought that toxicity of osimertinib would be clearly favorable in comparison with conventional EGFR-TKI with low selectivity.

As evidence for the clinical effect relevant to osimertinib, based on the results of the AURA phase I study and the 2 AURA phase II studies, overall response rate (ORR) and duration of response (DoR) of osimertinib as 1st-line treatment was reported to be 75% (95% confidence interval [CI]: 62–85) and 18 months, respectively.^[4]^ In addition, in the 2 studies still in progress (AURA extension study and AURA2 study), superior efficacy was also reported in T790M-positive patients having treatment history.

In the AURA extension study, ORR was 61% (95% CI: 54–68) and median DoR and median progression-free survival (PFS) were not calculable (NC); and in AURA2 study, ORR, median DoR, and median PFS were 71% (95% CI: 64–77), 7.8 months (95% CI: 7.1 months to ∼NC months), and 8.6 months (95% CI: 8.3–9.7), respectively.^[5]^

The main adverse events reported in the group receiving 1st-line treatment in the AURA study, AURA extension study, and AURA2 study were rash (all grades: 77%, ≥grade 3: 2%; all grades: 40%, ≥grade 3: 1%; all grades: 42%, ≥grade 3: 1%) and diarrhea (all grades: 73%, ≥grade 3: 3%; all grades: 45%, ≥grade 3: 1%; all grades: 39%, ≥grade 3: 1%), respectively.^[4,5]^ These results suggest the possibility of relatively safe use of osimertinib with few serious adverse events for the elderly in comparison with conventional EGFR-TKI, while showing an antitumor effect for T790M-positive patients. However, it is difficult to show sufficient data for these estimations.

At this time, we are prospectively investigating the efficacy and safety of osimertinib for elderly patients ≥75 years old with ineffective prior EGFR-TKI treatment or with recurrence in EGFR-TKI mutation-positive and T790M mutation-positive nonsmall-cell lung cancer (NSCLC).

On the other hand, tolerance sometimes soon develops in patients in whom treatment with EGFR-TKI was effective, and it is said that about half of the tolerance is caused by the resistant mutation T790M. However, since histological tissue examination of lung cancer is more highly invasive than for cancers of other organs, there are many difficult cases of patients with exacerbation after treatment involving particularly frequent re-tests. The expression of T790M in plasma DNA using the highly sensitive polymerase chain reaction appears in a report to relate to resistant generation of EGFR-TKI, but there are still many questions about the concordance rate with tissue T790M. We are therefore prospectively investigating positive/negative concordance rate of T790M between tumor tissue or humoral specimen and plasma DNA in the present study.

### 1.1 Endpoints

#### 1.1.1 Primary

The primary endpoint is ORR.

#### 1.1.2 Secondary

The secondary endpoints are PFS, overall survival (OS), disease control rate (DCR), safety, and positive/negative concordance rate of T790M between tumor tissue or humoral specimen and plasma DNA.

## 2 Methods and analysis

### 2.1 Study design

**Figure 1:**
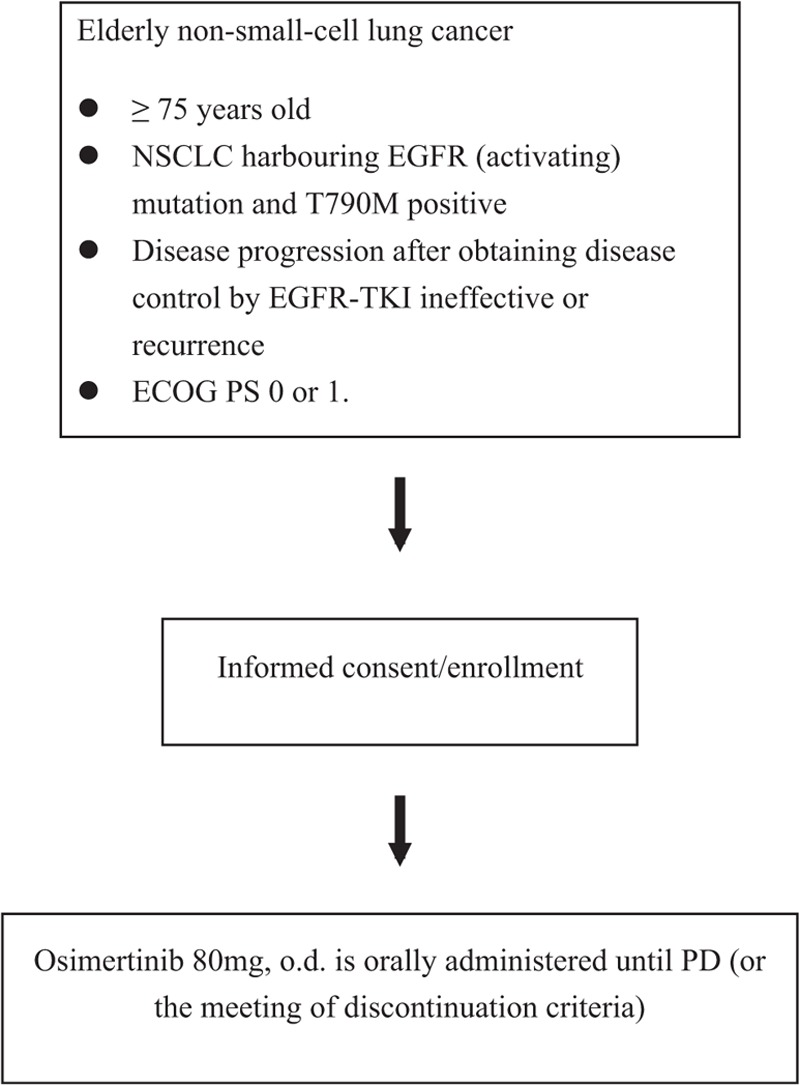
Study flow chart.

The study is single arm, open trial. Figure 1 depicts a flow chart of the study.

### 2.2 Study setting

Forty-seven hospitals agreed to take part in this study. The protocol was approved by the institutional review board of each hospital. Written informed consent was obtained from all patients before registration, in accordance with the Declaration of Helsinki. Patients are registered in this study after independent review by the Data Center of the Clinical Research Support Centre Kyushu, where the potential subjects are screened against the inclusion and exclusion criteria. At least annual independent monitoring is planned, in accordance with the Japanese clinical trial guideline.

### 2.3 Participants

The inclusion criteria are as follows:

(1) Patients with histologically or cytologically confirmed stage IIIB/IV NSCLC and postoperative recurrence.
(2) Patients with recurrence after the effect of higher than stable disease (SD) or not effective (less than SD) was observed in best overall response according to RECIST by treatment of EGFR-TKI (e.g., such as erlotinib, gefitinib, afatinib) for NSCLC.
(3) NSCLC harboring EGFR (activating) mutation and T790M positive.
(4) Patients able to accept treatment with oral medicine.
(5) Patients having at least 1 measurable lesion according to RECIST criteria.
(6) Performance Status (ECOG) 0 to 1.
(7) In principle, patients capable of participating this study for at least 2-week hospital stay or corresponding management.
(8) Patients who are ≥75 years of age (at the time of enrollment).
(9) Patients for whom bone marrow, hepatic, and renal functions have all been confirmed as normal within 14 days prior to enrollment according to the following clinical test standards:
  (1) white blood cell ≥ 3000/mm^3^ to ≤ 12,000/mm^3^
  (2) neutrophil count ≥ 1500/mm^3^
  (3) platelet count ≥ 100,000/mm^3^
  (4) hemoglobin ≥ 9.0 g/dL
  (5) aspartate aminotransferase, alanine aminotransferase ≤ 100 IU/L
  (6) total bilirubin ≤ 1.5 mg/dL
  (7) creatinine ≤ 2.0 mg/dL
  (8) SpO_2_ (Room air) ≥ 90%
(10) Patients with life expectancy of at least 3 months.
(11) Patients whose prior treatment has passed by the planned starting point of administration (eligible from the same day after past period).
  (1) Chemotherapy: ≥4 weeks have passed since the final treatment of prior chemotherapy
  (2) EGFR-TKI: the next day after the last administration
  (3) Radiation therapy in the case of chest: ≥12 weeks have passed since the day of final radiation treatment
  (4) In the case of radiation to other than chest: ≥2 weeks have passed since the day of final radiation treatment
  (5) Operation/treatment (including chest drainage): ≥4 weeks have passed since the day of final operation/treatment
(12) Patients who provide written informed consent to participate in the study

The exclusion criteria are as follows:

(1) Patients who have treatment history of osimertinib or other 3rd-generation EGFR-TKI.
(2) Patients with pulmonary disorders such as idiopathic pulmonary fibrosis, interstitial pneumonia, pneumoconiosis, active radiation pneumonitis, and drug-induced pneumonia.
(3) Patients with infectious disorder requiring intravenous injection of antibacterial drugs or antimycotics.
(4) Patients unable to swallow oral medications.
(5) Patients currently receiving (or unable to stop use prior to receiving the first dose of study treatment) medications or herbal supplements known to be potent inhibitors of CYP3A4 (at least 1 week prior) and potent inducers of CYP3A4 (at least 3 weeks prior). All patients must avoid concomitant use of any medications, herbal supplements, and/or ingestion of foods with known inducer/inhibitory effects on CYP3A4.
(6) Patients currently receiving checkpoint inhibitor.
(7) Patients with any of the following cardiac criteria:
  (1) Mean resting corrected QT interval (QTc using Fridericia formula) >470 ms.
  (2) Any clinically important abnormalities in rhythm, conduction, or morphology of resting ECG (e.g., complete left-bundle branch block, 3rd-degree heart block, 2nd-degree heart block).
  (3) Any factors that increase the risk of QTc prolongation or risk of arrhythmic events such as heart failure, hypokalemia, congenital long QT syndrome, family history of long QT syndrome, or unexplained sudden death under 40 years of age in 1st-degree relatives or any concomitant medication known to prolong the QT interval.
(8) Patients who are pregnant, nursing, or possibly pregnant.
(9) Patients with symptomatic brain metastasi.
(10) Patients with active double cancer.
(11) Patients with uncontrollable diabetes mellitus.
(12) Patients who have complications of clinical concern.
  (Such as uncontrollable cardiac disease, severe cardiac arrhythmia requiring medical treatment, sustained serious diarrhea.)
(13) Any other patients who are regarded as unsuitable for this study by the investigators.

### 2.4 Dose and treatment regimens

Osimertinib 80 mg OD tablet is orally administered. Oral administration of osimertinib is continued until progressive disease or the criteria for discontinuation.

### 2.5 Rationale for the setting of the number of enrolled subjects

From data of the 2 AURA phase II studies (AURA extension study and AURA2 study) in EGFR-T790M mutation-positive patients, ORR was reported to be 61% (95% CI: 54–68) in the AURA extension study (n = 201), and ORR and median PFS were reported to be 71% (95% CI: 64–77) and 8.6 months (95% CI: 8.3–9.7) in AURA2 study (n = 210), respectively.^[4,5]^ In addition, regarding docetaxel, assumed the standard treatment in elderly patients in Japanese guidelines, ORR of docetaxel was reported to be 22.7% in a controlled study with vinorelbine and 41.2% in a study of combined administration of carboplatin and pemetrexed in elderly Japanese patients.^[6,7]^ The expected response rate and threshold response rate are determined to be 60% and 35%, respectively. Under these conditions, when a 2-sided significance level of 5% and a test power of 80% are assumed, 31 subjects are required. Considering allowance for dropouts, 35 subjects were planned.

### 2.6 Statistical methods

ORR: response rate and its 2-sided 95% CI (Wilson method). When the lower limit of the estimated CI exceeds a threshold of 35%, statistical significance is decided.

PFS: survival curve, median (Kaplan–Meier method), CI of the median (Brookmeyer and Crowley method), standard error of the annual rate (Greenwood method).

OS: survival curve, median (Kaplan–Meier method), CI of the median (Brookmeyer and Crowley method), standard error of the annual rate (Greenwood method).

DCR: disease control rate and its 2-sided 95% CI (Wilson method).

Concordance rate of T790M: sensitivity, specificity, positive predictive value, negative predictive value, accuracy, and kappa coefficient.

Safety: grade and frequency of each adverse event.

### 2.7 Population to be analyzed

All subjects enrolled in this study (full analysis set [FAS]); the subjects excluding the patients with serious violations (such as serious protocol deviation, violation for inclusion/exclusion criteria, and violation for prohibited concomitant medication/therapy) from FAS (per protocol set); and among the FAS in which the protocol treatment is provided at least once (safety analysis set).

### 2.8 Ethics

The trial received ethical approval from the Ethics Committee of Kyoto Prefectural University of Medicine, Kyoto, Japan (number: ERB-C-630-3, the last edition ver 3. December 21, 2016). The trial is subject to the supervision and management of the Ethics Committee.

### 2.9 Trial status

This study opened to recruitment in March 2016, with a planned last follow up in October 2019. As of April 2018, 34 subjects have been enrolled.

## 3 Discussion

It is now well established that acquisition of a second mutation in EGFR, resulting in substitution of threonine at the “gatekeeper” amino acid 790 to methionine (T790M), is the most common resistance mechanism. Second-generation irreversible EGFR-TKIs such as afatinib are effective in untreated EGFR-mutant lung cancer. However, as monotherapy, they have failed to overcome T790M-mediated resistance in patients, because concentrations at which these irreversible TKIs overcome T790M activity preclinically are not achievable in humans due to dose-limiting toxicity related to nonselective inhibition of wild-type EGFR. This situation has led to the development of “3rd-generation” EGFR-TKIs that are designed to target T790M and EGFR-TKI-sensitizing mutations more selectively than wild-type EGFR. Osimertinib has been shown to be effective against both EGFR tyrosine kinase inhibitor-sensitizing and T790M resistance mutations. On the other hand, an increase in life expectancy in the general population has led to a rise in the incidence of lung cancer in elderly patients. In the United States, almost half (47%) of all lung cancer patients are more than 70 years old, and 14% are more than 80 years old. By the same token, in Japan, the number of elderly patients diagnosed with lung cancer is increasing, with almost half of all Japanese patients with NSCLC reported as 75 years of age or older. We therefore need to evaluate efficacy and safety in elderly patients.

## Acknowledgments

The authors thank the patients, their families, and all investigators involved in this recent study.

## Author contributions

**Conceptualization:** Junji Uchino, Akira Nakao, Masaki Fujita.

**Formal analysis:** Kenichi Yoshimura.

**Funding acquisition:** Junji Uchino.

**Investigation:** Junji Uchino, Akira Nakao, Nobuyo Tamiya, Yoshiko Kaneko, Tadaaki Yamada.

**Project administration:** Junji Uchino, Akira Nakao, Nobuyo Tamiya, Yoshiko Kaneko, Tadaaki Yamada.

**Supervision:** Masaki Fujita, Koichi Takayama.
